# The bidirectional relationship between diabetes and poor muscle function in older adults: data from two population-based studies

**DOI:** 10.1093/gerona/glag004

**Published:** 2026-01-14

**Authors:** Caterina Trevisan, Francesca Remelli, Stefania Bandinelli, Maria C Corti, Giuseppe Sergi, Davide Atti, Marianna Noale, Stefania Maggi, Jack M Guralnik, Luigi Ferrucci, Stefano Volpato

**Affiliations:** Department of Medical Sciences, University of Ferrara, Ferrara, Italy; Orthogeriatrics Unit, University Hospital Sant’Anna of Ferrara, Ferrara, Italy; Aging Research Center, Department of Neurobiology, Care Sciences and Society, Karolinska Institutet, Stockholm, Sweden; Department of Medical Sciences, University of Ferrara, Ferrara, Italy; Aging Research Center, Department of Neurobiology, Care Sciences and Society, Karolinska Institutet, Stockholm, Sweden; Geriatric Unit, Azienda Toscana Centro, Florence, Italy; Epidemiology Division, Azienda Zero, Padua, Italy; Department of Medicine DIMED, University of Padua, Padua, Italy; Department of Medical, Surgical and Health Sciences, University of Trieste, Trieste, Italy; Institute of Neuroscience, National Research Council, Padua, Italy; Institute of Neuroscience, National Research Council, Padua, Italy; Department of Epidemiology and Public Health, University of Maryland School of Medicine, Baltimore, Maryland, United States; Translational Gerontology Branch, National Institute on Aging, National Institutes of Health, Baltimore, Maryland, United States; Department of Medical Sciences, University of Ferrara, Ferrara, Italy; Orthogeriatrics Unit, University Hospital Sant’Anna of Ferrara, Ferrara, Italy; (Medical Sciences Section)

**Keywords:** Aged, Diabetes, Physical disability, Sarcopenia

## Abstract

**Background:**

Skeletal muscle dysfunction contributes significantly to disability, which is one of the most common complications of diabetes in older adults. We aimed to assess whether diabetes was associated with a steeper muscle strength decline and whether lower strength is related to a higher diabetes incidence in older adults.

**Methods:**

A prospective analysis of data from two Italian population–based studies in older adults (the Invecchiare in Chianti and Progetto Veneto Anziani studies). Diabetes was assessed at baseline and after a median of 4.4 (first follow-up) and 6.3 years (second follow-up) using multiple sources of information. Muscle function was assessed as handgrip strength.

**Results:**

The sample comprised 3927 participants (58.6% females) with a mean age of 75.5 years (29.6% aged ≥80 years). After adjusting for potential confounders, the decline in muscle strength among individuals with diabetes exceeded that of those without diabetes by 0.70 kg (95% CI, −1.30 to −0.11) at the first follow-up and by 0.84 kg (95% CI, −1.61 to −0.07) at the second follow-up. In those taking oral antidiabetics, this association was even stronger. Over a median 5-year follow-up, 186 incident diabetes cases were recorded. In a multivariable Cox regression, each 1-SD higher in the handgrip/body weight ratio was associated with an 20% lower likelihood of incident diabetes (95% CI, 0.68-0.95, *n* = 3102).

**Conclusions:**

These findings demonstrate an independent circular relationship between diabetes and skeletal muscle strength. In older people, muscle dysfunction may be a long-term diabetes complication. Whether increasing muscle strength might reduce diabetes risk remains to be determined.

## Introduction

Diabetes is one of the most common chronic conditions of older people, affecting globally one out of five individuals aged 65-99 years. This number is expected to increase even more, considering that in aging societies, the incidence of diabetes is constantly growing while the absolute rates of mortality have declined. In fact, it has been estimated that the number of patients with diabetes aged 65-99 years will reach 195.2 million by 2030 and 276.2 million by 2045.^1^

One of the most relevant clinical aspects of diabetes in older persons is its association with an increased risk of geriatric syndromes such as functional impairment and physical disabi­lity, falls, and frailty, which are common conditions that heavily impact quality of life and the need for assistance from health and social services.^2^ Increasing evidence supports the existence of a strong relationship between diabetes and functional impairment,^3^ and a meta-analysis demonstrated that diabetes increased the risk of mobility disability and dependency in basic and instrumental daily activities by roughly 51%, 65%, and 82%, respectively.^4^

Although comorbidities related to diabetes play a substantial role in the relationship between diabetes and the risk of disability,^5^ they do not thoroughly explain such an association. In this context, skeletal muscle dysfunction—defined as poor muscle quality and strength—may be one of the mechanisms explaining the risk of physical impairment associated with diabetes.^6^ Over the last few decades, several studies have shown an association between diabetes and impairment of carbohydrate metabolism with poorer muscle quality.^7^ However, these data should be considered with caution because most of these analyses relied on cross-sectional studies,^7–9^ with only few studies reporting results from longitudinal cohorts.^10–12^ Furthermore, there is still insufficient information on the older population.

The interpretation of the biological mechanisms linking diabetes and muscle dysfunction is made difficult by recent evidence suggesting that low muscle strength might precede and predict the onset of type 2 diabetes. Indeed, skeletal muscle is a major determinant of glucose metabolism and serum glucose uptake, and muscle strength, a marker for muscle mass and quality, is inversely associated with insulin resistance.^13^ From this point of view, age-related sarcopenia might favor the onset of insulin resistance and diabetes development, but this hypothesis has not been formally tested in older persons.^14^

This study aimed to assess the bidirectional relationship between diabetes and muscle strength in older adults. In particular, we evaluated whether people with diabetes have a steeper handgrip (HG) strength decline over time compared to those without diabetes and, in turn, if older individuals without diabetes with lower HG have an increased risk of developing diabetes.

## Methods

### Study population

A prospective analysis of data collected from two large population–based cohort studies was performed. This analysis was proceeded by a retrospective harmonization of data from the InCHIANTI (Invecchiare in Chianti)^15^ and Pro.V.A. (Progetto Veneto Anziani)^16^ studies.

The InCHIANTI study is a prospective, population–based study of older people conducted by the Laboratory of Clinical Epidemiology of the *Istituto Nazionale di Riposo e Cura per Anziani* (INRCA) and aimed at identifying risk factors for late-life disability. Participants were randomly selected by the city registers of two towns in the Chianti Valley (Tuscany, Italy): Greve del Chianti and Bagno a Ripoli. The baseline assessment was performed in 1998-2000 and involved 1453 subjects (aged 20-102). For the present study, we considered data from the baseline, 3 and 6-year follow-up assessments of the InCHIANTI study population.

The Pro.V.A. is a prospective, population–based study whose primary aim was to explore the mechanisms through which chronic diseases may lead to the development of disability in older people, with a special focus on cardiovascular and osteoarticular conditions. Baseline assessment took place between 1995 and 1998, and included 3099 individuals aged ≥ 65 years, randomly selected from health district lists of two towns of north-east Italy—Rovigo and Camposampiero—without exclusion criteria, and applying a stratification by age and sex. Active follow-up assessments were performed after 4 and 7 years from baseline.

The harmonization process was conducted retrospectively following international recommendations,^17^ through five main steps: (1) Definition of the research question and study objectives; (2) Identification of the core variables needed to verify the hypotheses, their availability and methods of assessment in the two studies; (3) Assessment of the distribution of variables selected in the single studies; (4) Documentation of the study-specific data items processed under common formats to produce the harmonized variables; (5) Comparison of the distribution of newly harmonized variables between the two study cohorts, to assess the consistency of data.

For our main analysis, from the original pooled cohort of 4552 participants, we excluded 298 individuals younger than 65 years, and 327 with no available HG assessments over the follow-up, reaching a final analytical sample of 3927 older adults. From the comparison of the characteristics of individuals included in the final sample and those excluded due to missing HG measurement (Table S1), we found that the latter group was more likely to be older, widowed, and physically sedentary. Moreover, participants excluded from the analytical sample had lower body mass index (BMI), poorer functional and cognitive performance, and a higher prevalence of most chronic conditions, except for diabetes, osteoarthritis, chronic obstructive pulmonary disease (COPD), and cancer.

When evaluating the association between HG strength and incident diabetes, from the initial analytical sample of 3927 participants, we excluded 607 participants with diabetes at baseline, 151 with missing data on HG (*n* = 108 from the InCHIANTI, and *n* = 43 from the Pro.V.A. study), 35 with missing data on baseline body weight (*n* = 3 from the InCHIANTI, and *n* = 32 from the Pro.V.A. study), and 32 with missing data on both baseline HG and body weight (*n* = 24 from the InCHIANTI, and *n* = 8 from the Pro.V.A. study). The final analytical subsample included 3102 individuals.

The InCHIANTI study protocol was approved by the ethical committee of the Italian National Institute of Research and Care on Aging. The Pro.V.A. study protocol was approved by the Ethics Committees of the University of Padova and of the nrs. 15 and 18 Local Health Units of the Veneto Region. All participants gave their written informed consent to participate in the study.

### Data collection

The following information was collected and harmonized for each participant from the two study populations:

#### Sociodemographic data

Information on participants’ age, sex, marital status, and educational level was recorded through personal interviews by trained nurses and physicians. Education was classified as none or elementary (≤5 years), middle or high school (6-13 years), and university or more (≥14 years), considering the cut-offs of the Italian school system.

#### Lifestyle characteristics

Based on smoking habits, participants were categorized as never, former, or current smokers. Alcohol consumption was classified as no (0 alcohol units [AU] per week), mild (<7 or <14 AU per week for females and males, respectively), and high (≥7 or ≥14 AU per week for females and males, respectively) consumers. Both in the InCHIANTI and the Pro.V.A. studies, participants’ physical activity level was assessed through structured questionnaires recording the frequency and intensity of engagement in various activities, expressed as hours per week.^18^^,^^19^ We classified physical activity level as: low, if participants were completely inactive or engaged only in light intensity physical activity (eg, gardening, walking, dancing) not more than 1 hr/week; and moderate-to-high, if participants performed light physical activity at least 2 hr per week or moderate intensity physical activities (eg, running, swimming, exercising in a gym) at least 1 hr per week.

#### Health-related information

Anthropometric measures, including body weight, height, and waist circumference, were objectively assessed, and the BMI (kg/m^2^) was computed from the ratio between weight and height squared. As part of the comprehensive geriatric assessment, functional status was assessed by self-report of ability to perform activities of daily living (ADL) and instrumental ADL (IADL) survey tools,^20^ while cognitive performance was evaluated by the Mini-Mental State Examination (MMSE).^21^ Depressive mood was rated using the Center for Epidemiological Studies Depression (CES-D) scale in the InCHIANTI study and the 30-item Geriatric Depression Scale (GDS) in the Pro.V.A. study. The presence of depressive symptoms was established based on a score ≥16 at the CES-D (sensitivity: 0.87 [95% CI, 0.82-0.92]; specificity 0.70 [95% CI, 0.65-0.75]^22^) or ≥11 at the GDS (sensitivity 0.828 [95% CI, 0.813-0.831], specificity 0.705 [95% CI, 0.703-0.706]^23^). These tools have demonstrated similar accuracy in detecting depressive symptoms, with a slightly higher sensitivity for the GDS-30 and a higher specificity for the CES-D, but close positive and negative predictive values and misclassification rate.^23^ The baseline prevalence of specific medical conditions was established using standardized criteria that combined information from self-reported history, medical records, and a clinical medical examination. Diagnostic algorithms were modified versions of those used in the Women’s Health and Aging Study.^24^ The following diseases were assessed: arterial hypertension, cardiovascular diseases (including at least one among angina, myocardial infarction, heart failure, peripheral artery disease, and stroke), COPD, cancer, osteoarthritis (at knee or hip), and previous hip fracture.

#### Assessment of diabetes

Diabetes was assessed through medical records (previously diagnosed diabetes and/or use of antidiabetic medications) or, among individuals with undiagnosed diabetes, by fasting plasma glucose ≥126 mg/dL, according to the American Diabetes Association guidelines.^25^ Moreover, fasting plasma glucose values were considered in our analysis as an indirect indicator of glycemic control. Based on the onset of diabetes, we distinguished people with a diagnosis of diabetes at baseline (*prevalent diabetes*) and participants who developed diabetes during the follow-up (*incident diabetes*). Antidiabetic drugs, ie, oral antidiabetics (metformin, sulfonylureas, glinides) and insulin, were recorded at baseline through structured interviews and review of medical documentation.

#### Handgrip strength

In both the InCHIANTI and Pro.V.A. studies, HG strength was measured with JAMAR hand-held dynamometers (Irvington, NY for the InCHIANTI study, and Fred Sammons, Inc., IL for the Pro.V.A. study) and expressed in kilograms. The grip strength of each hand was assessed twice; the highest result of the obtained measures was used in our analysis. Individuals with pain or who had undergone any surgery on the hand were excluded from the assessment of the HG.^26^^,^^27^

### Statistical analysis

The characteristics of the study participants were expressed as means and standard deviations (SD) for quantitative variables and as absolute and relative frequencies for the categorical ones. Comparison of baseline characteristics by prevalent or incident diabetes and by study cohort was performed through ANOVA or Chi-squared test, as appropriate.

The association of diabetes with changes in HG over time was assessed through linear mixed models, setting a random intercept, and including the main effects and the interaction between diabetes and time (as a categorical variable, distinguishing baseline [reference], the first [T1] and second [T2] assessments). In particular, two main exposures were considered. First, diabetes was included in the model as a time-varying variable; second, the presence of diabetes at baseline and use of antidiabetic therapy were categorized into four groups (ie, no diabetes, diabetes not under pharmacologic therapy, diabetes under oral antidiabetics only, and diabetes under insulin). Adjustment for potential confounding factors was performed. In particular, Model 1 was adjusted for study cohort, age, and sex; Model 2 was further adjusted for educational level, smoking habits, alcohol consumption, physical activity level, MMSE, osteoarthritis, COPD, cancer, and depressive symptoms; and, in Model 3, the presence of cardiovascular diseases (as a time-varying variable) was also included. The strength of these associations was described with β coefficients and 95% CI. Interactions between diabetes and sex were also formally tested in multivariable models.

The association between HG strength and incident diabetes was tested in the subsample of participants free from diabetes at baseline with available HG and body weight measurements at baseline (*n* = 3102). For this analysis, after checking compliance with the proportional hazard assumption, we employed Cox regression models. As the main exposure, in accordance with the current literature,^14^ we considered the ratio of HG to body weight, which was transformed into sex-specific *z*-scores (mean handgrip to body weight ratio: 0.47 [SD 0.13] for men, and 0.33 [SD 0.11] for women) to facilitate results interpretation. The outcome was incident diabetes; the time to incident diabetes was computed considering the interval between the baseline and the follow-up assessment when the diabetes diagnosis was recorded (for incident diabetes cases) or censored to the last available follow-up or death, whichever occurred first. The strength of these associations was described as hazard ratio (HR) and 95% CI. Models were adjusted for study cohort, age, sex (Model 1), educational level, smoking habits, alcohol consumption, physical activity level, depressive symptoms (Model 2), and cardiovascular diseases at baseline (Model 3). Possible non-linearity of the association between HG and the risk of diabetes over the follow-up was checked by including a penalized spline term for the ratio HG/body weight in the model. This analysis showed that the *p*-value for the spline term was non-significant (*p* = .36), suggesting that the relationship could be assumed to be approximately linear. The fully adjusted association of the HG/body weight ratio against HR of diabetes was graphically illustrated in a plot with a logarithmic scale of HR on the *y* axis.

Stratified analyses by sex and by study cohort were performed to evaluate possible effect modification of these factors on the study estimates. Finally, to verify whether the association between muscle strength and incident diabetes was independent of baseline glycemia, as an indicator of glycemic control, we performed sensitivity analyses by including this variable in the fully adjusted model.

All statistical tests were two-tailed and considered a *p*-value <.05 as statistically significant. The R software version 4.3.3 was used for statistical analyses (R Foundation for Statistical Computing, Vienna, Austria).

## Results

The study sample comprised 3927 participants (58.6% females) with a mean age of 75.5 years (29.6% aged ≥80 years), whose characteristics are shown in Table 1. Only 15.5% of individuals had at least completed middle school, 54.2% were partnered, and 37.6% were widowed. At the baseline, 607 (15.7%) individuals had diabetes. Of these, 241 (39.7%) were treated only with oral antidiabetic drugs (10 [1.6%] treated with only biguanides, 133 [21.9%] with sulphanilureas, 89 [14.7%] with combined biguanides and sulphanilureas, and 9 [1.5%] with alpha-glucosidase inhibitors, alone or in association with other oral antidiabetics), while 48 (7.9%) were under insulin therapy (either in combination or not with oral antidiabetics). When comparing the two cohorts (Table S2), participants in the InCHIANTI were slightly younger (mean age 74.5 vs 75.6 years, *p* < .001) and more likely to have higher scholarity (25.9% vs 11.9% had at least a middle school degree, *p* < .001), better functional and cognitive status, and, on average, a higher HG at baseline (28.57 vs 25.98 kg, *p* < .001). Moreover, the InCHIANTI participants showed lower frequencies of physical inactivity (19.8% vs 34.6%, *p* < .001), hypertension, cardiovascular diseases, COPD, and diabetes (12.6% vs 16.5%, *p* = .004).

**Table 1.** Baseline characteristics of the pooled cohort by the presence of diabetes over the follow-up.

	Overall	No diabetes	Prevalent diabetes	Incident diabetes	*p*-Value
***n***	3927 (100)	3134 (79.8)	607 (15.5)	186 (4.7)	
**Age (years)**	75.46 (7.45)	75.43 (7.52)	75.93 (7.19)	74.45 (6.96)	.052
**Female sex**	2302 (58.6)	1826 (58.3)	357 (58.8)	119 (64.0)	.305
**Educational level**					.197
** None or elementary**	3317 (84.5)	2626 (83.8)	531 (87.5)	160 (86.0)	
** Middle or High school**	528 (13.4)	441 (14.1)	64 (10.5)	23 (12.4)	
** University or above**	82 (2.1)	67 (2.1)	12 (2.0)	3 (1.6)	
**Marital status**					.077
** Never married, single**	299 (7.6)	257 (8.2)	32 (5.3)	10 (5.4)	
** Partnered**	2126 (54.2)	1699 (54.2)	318 (52.4)	109 (58.6)	
** Separated/divorced**	24 (0.6)	17 (0.5)	6 (1.0)	1 (0.5)	
** Widowed**	1475 (37.6)	1159 (37.0)	250 (41.2)	66 (35.5)	
**Smoking habits**					.072
** Never smokers**	2367 (60.3)	1881 (60.0)	369 (60.9)	117 (62.9)	
** Former smokers**	1177 (30.0)	927 (29.6)	195 (32.2)	55 (29.6)	
** Current smokers**	381 (9.7)	325 (10.4)	42 (6.9)	14 (7.5)	
**Alcohol consumption**					<.001
** No**	2298 (58.7)	1802 (57.6)	399 (66.1)	97 (52.7)	
** Moderate**	903 (23.0)	738 (23.6)	109 (18.0)	56 (30.4)	
** High**	717 (18.3)	590 (18.8)	96 (15.9)	31 (16.8)	
**Low physical activity**	1194 (30.7)	921 (29.8)	222 (36.8)	51 (27.4)	.002
**Body Mass Index (kg/m^2^)**	27.57 (4.46)	27.13 (4.23)	29.19 (4.76)	29.72 (5.41)	<.001
**Waist circumference (cm)**	95.6 (11.3)	94.4 (11.0)	100.1 (11.2)	99.9 (11.6)	<.001
**ADL score**	5.29 (1.20)	5.32 (1.17)	5.06 (1.39)	5.45 (0.91)	<.001
**IADL score**	6.34 (2.19)	6.39 (2.17)	6.01 (2.36)	6.64 (1.79)	<.001
**MMSE**	23.85 (5.32)	23.87 (5.37)	23.36 (5.35)	24.92 (4.07)	.002
**Depressive symptoms**	1217 (33.0)	952 (32.4)	210 (37.3)	55 (30.2)	.054
**Arterial hypertension**	2790 (71.1)	2168 (69.2)	478 (78.7)	144 (77.4)	<.001
**Cardiovascular diseases**	1066 (27.1)	788 (25.1)	232 (38.2)	46 (24.7)	<.001
**Previous hip fracture**	134 (3.4)	104 (3.3)	19 (3.1)	11 (5.9)	.154
**Osteoarthritis**	1251 (31.9)	978 (31.2)	207 (34.1)	66 (35.5)	.207
**COPD**	421 (10.7)	331 (10.6)	73 (12.0)	17 (9.2)	.446
**Cancer**	298 (7.6)	231 (7.4)	51 (8.4)	16 (8.6)	.579
**Handgrip (kg)**	26.59 (10.19)	26.68 (10.18)	25.97 (10.34)	27.11 (9.77)	.241

Abbreviations: ADL, activities of daily living; COPD, chronic obstructive pulmonary disease; IADL, instrumental activities of daily living; MMSE, Mini-Mental State Examination

Missing values in marital status (*n* = 3), smoking habits (*n* = 2), alcohol consumption (*n* = 9), depressive symptoms (*n* = 243), ADL (*n* = 10), IADL (*n* = 14), Body Mass Index (*n* = 122), MMSE (*n* = 30), arterial hypertension (*n* = 2), hip fracture history (*n* = 6), cancer (*n* = 2), COPD (*n* = 2).

When comparing individuals free from diabetes and those with prevalent and incident diabetes (Table 1), we found that those affected by diabetes at baseline (prevalent diabetes) were less likely to consume alcohol and be physically active; moreover, this group had, on average, higher BMI and higher prevalence of hypertension, cardiovascular diseases, and worse functional and cognitive performance. Comparing diabetes and non-diabetes groups, we found no statistically significant differences in HG baseline, although participants with prevalent diabetes tended to have lower grip strength than those without diabetes at baseline.

**Figure 1.**
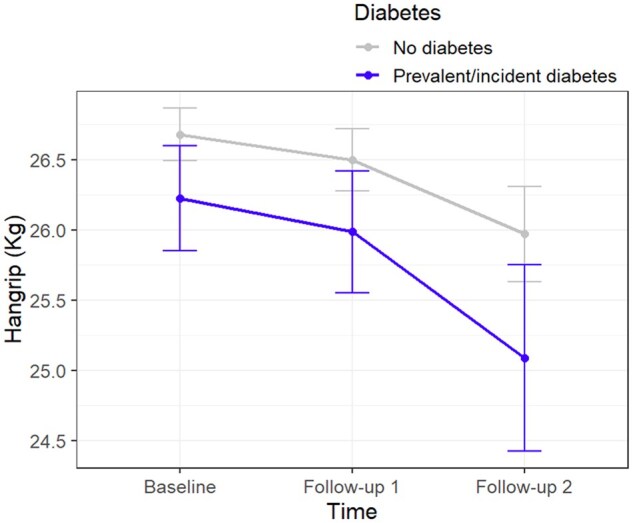
Mean handgrip values in the pooled cohort by diabetes status during the observation period. *Note*. At each time point, bars represent standard errors.

Over the follow-up, individuals with baseline diabetes tended to have a steeper decline in muscle strength than those without diabetes (Figure 1), and this trend was confirmed using linear mixed models. Specifically, compared with individuals without diabetes, those with diabetes showed an additional average reduction in HG strength of 0.68 kg (95% CI, −1.27 to −0.08) at the first follow-up (median 4.41 years from baseline; interquartile range [IQR]: 3.26-4.91) and 0.79 kg (95% CI, −1.56 to −0.01) at the second follow-up (median 6.29 years from baseline; IQR: 6.06-6.95; Table 2). Adjustment for covariates slightly attenuated these estimates. Stronger effect sizes were observed in women than in men, although the differences were not statistically significant (*p* for interaction with sex >0.10) (Table S3). When stratified by study cohort, the results were attenuated; however, a similar trend for HG changes over time, as a function of diabetes status, was still observed (Table S4).

**Table 2.** Linear mixed model for the association between diabetes and changes in handgrip over the follow-up in the sample of 3927 individuals.

	β Coefficient (95% CI), *p*-Value					
	Model 1	*p*	Model 2	*p*	Model 3	*p*
**Diabetes as a time-varying variable**						
**Diabetes (intercept, T0)**	0 (−0.56, 0.56)	.989	0.14 (−0.41, 0.70)	0.616	0.25 (−0.31, 0.80)	.385
Diabetes*T1	**−0.68 (−1.27, −0.08)**	**.026**	**−0.74 (−1.34 to −0.14)**	**0.015**	**−0.70 (−1.30 to −0.11)**	**.021**
Diabetes*T2	**−0.79 (−1.56, −0.01)**	**.046**	**−0.86 (−1.64 to −0.09)**	**0.029**	**−0.84 (−1.61 to −0.07)**	**.034**
**Baseline diabetes and antidiabetic therapy** **(ref: no diabetes)**						
***Intercept (T0)***						
**Diabetes with no drug therapy (*n* = 318)**	0.34 (−0.46 to 1.14)	.405	0.31 (−0.48 to 1.09)	0.443	0.42 (−0.37 to 1.20)	.298
**Diabetes under oral antidiabetics (*n* = 241)**	−0.34 (−1.25 to 0.57)	.464	−0.02 (−0.92 to 0.87)	0.958	0.09 (−0.81 to 0.98)	.850
**Diabetes under insulin (*n* = 48)**	**−2.78 (−4.75 to −0.81)**	**.006**	−1.71 (−3.70 to 0.28)	0.093	−1.46 (−3.45 to 0.52)	.149
***Interaction with time***						
Diabetes with no drug therapy*T1	−0.75 (−1.57, 0.07)	.072	−0.77 (−1.59 to 0.05)	0.065	−0.79 (−1.61 to 0.02)	.057
Diabetes with no drug therapy*T2	−1.08 (−2.21, 0.06)	.063	−1.03 (−2.16 to 0.11)	0.076	−1.07 (−2.20 to 0.06)	.065
Diabetes under oral antidiabetics*T1	−0.74 (−1.68, 0.21)	.127	−0.75 (−1.70 to 0.19)	0.119	−0.65 (−1.59 to 0.29)	.177
Diabetes under oral antidiabetics*T2	**−1.66 (−2.98, −0.34)**	**.014**	**−1.71 (−3.03 to −0.38)**	**0.012**	**−1.55 (−2.87 to −0.22)**	**.022**
Diabetes under insulin*T1	−0.41 (−2.66, 1.83)	.718	−0.5 (−2.74 to 1.75)	0.665	−0.27 (−2.51 to 1.97)	.813
Diabetes under insulin*T2	1.07 (−1.87, 4.02)	.475	0.89 (−2.04 to 3.82)	0.553	0.99 (−1.94 to 3.92)	.508

Abbreviations: T1, follow-up 1 (median: 4.41 [25th-75th percentiles: 3.26-4.91] years from baseline); T2, follow-up 2 (median: 6.29 [25th-75th percentiles: 6.06-6.95] years from baseline).

Model 1 is adjusted for study cohort, age, and sex. Model 2 is also adjusted for educational level, smoking habits, alcohol consumption, low physical activity, Mini-Mental State Examination, osteoarthritis, cancer, chronic obstructive pulmonary disease, and depressive symptoms. Model 3 is also adjusted for cardiovascular diseases (as a time-varying variable). Diabetes status is included in the model as a time-varying variable. The model considering baseline diabetes and antidiabetic therapy as main exposure was performed on 3884 participants. Bold font indicates significant *p*-values (<.05).

The longitudinal association between prevalent diabetes and change over time in HG strength was further investigated according to the type of antidiabetic therapy at baseline. Compared to patients without prevalent diabetes, those with diabetes using only oral agents had a steeper HG strength decline over time (-1.66 kg [95% CI, -2.98, -0.34] at the second follow-up), whereas patients using insulin therapy had lower strength at baseline, but a similar longitudinal HG trajectory as those without diabetes. Estimates did not substantially change after adjustment for potential confounders, including baseline fasting glucose level (data not shown).

**Figure 2.**
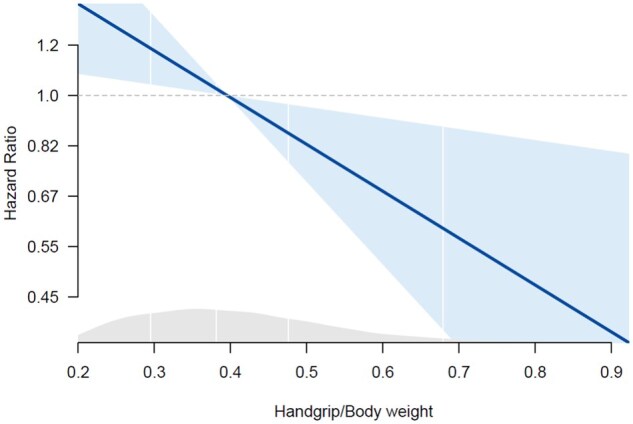
Association between handgrip/body weight values and the risk of diabetes during the follow-up. *Note*. The *y* axis is expressed on a logarithmic scale, as appropriate. The gray area indicates the density of each *x* value in the study sample. Estimates are derived from a Cox regression model adjusted for study cohort, age, sex, educational level, smoking habits, alcohol consumption, low physical activity, depressive symptoms, and cardiovascular diseases at baseline.

Over a median follow-up of 5.0 (interquartile range [IQR] 4.4-6.2) years, 186 (5.6%) new cases of diabetes were recorded (51 [5.7%] among the InCHIANTI and 135 [5.6%] in the Pro.V.A. cohort). Figure 2 shows the linear association between the HG/body weight ratio and incident diabetes, as estimated from Cox survival analysis. As reported in Table 3, after adjustment for multiple confounders, each 1-SD higher HG/body weight ratio was associated with a 21% lower probability of incident diabetes (95% CI, 0.67-0.93) with similar estimates in both sexes (interaction term not statistically significant). Results were confirmed after adjusting for potential confounders related to lifestyle and comorbidities (Table 3). However, a substantial attenuation was observed when including in the model also baseline fasting glycemia level as an indicator of glycemic control (HR = 0.88, 95% CI, 0.75-1.04, *p* = .14). The trend of the results was consistent in the two studies, although results were less evident in the InCHIANTI cohort (Table S5).

**Table 3.** Cox regression for the association between the baseline handgrip/body weight ratio and incident diabetes over the follow-up.

	Hazard ratio (95% CI) of diabetes, *p*-Value					
	Model 1	*p*	Model 2	*p*	Model 3	*p*
**All (*n* = 3102)**						
***Per each 1-SD HG/weight increase***	0.79 (0.67-0.93)	.004	0.80 (0.68-0.94)	0.007	0.80 (0.68-0.95)	.009
**Men (*n* = 1284)**						
***Per each 1-SD HG/weight increase***	0.79 (0.60-1.04)	.090	0.80 (0.60-1.05)	0.11	0.79 (0.60-1.05)	.10
**Women (*n* = 1818)**						
***Per each 1-SD HG/weight increase***	0.78 (0.64-0.96)	.017	0.78 (0.63-0.96)	0.02	0.77 (0.63-0.95)	.02

Abbreviations: HG, handgrip; SD, standard deviation.

Model 1 is adjusted for study cohort, age, and sex. Model 2 is also adjusted for educational level, smoking habits, alcohol consumption, low physical activity, and depressive symptoms. Model 3 is also adjusted for cardiovascular diseases at baseline. A 1-SD difference in the HG/body weight ratio corresponded to 0.13 in males, and 0.11 in females.

## Discussion

This study demonstrated a bidirectional, longitudinal, and independent association between type 2 diabetes and muscle strength in Italian older adults over a 7-year follow-up period. Using a pooled sample from two population–based studies, we demonstrated that diabetes is associated with a steeper decline in HG strength over time and that older participants with lower HG strength have an increased risk of incident diabetes during the follow-up. Estimates were consistent across sex and after adjustment for several potential confounding factors, suggesting an independent reciprocal relationship.

Our findings extend the results of previous studies on the relationship between diabetes and muscle health in older adults. Type-2 diabetes is a well-established risk factor for functional limitation and physical disability in older people^28^; several cross-sectional studies and systematic reviews with meta-analyses have consistently reported an association between type 2 diabetes with muscle mass and strength or sarcopenia as an underlying mechanism linking diabetes to physical disability.^7^^,^^29^^,^^30^ Nevertheless, only a few studies, with short follow-ups, demonstrated a greater decline in muscle mass or strength according to diabetes in older individuals.^12^ Our results suggest that older individuals with diabetes experienced an accelerated loss of muscle strength over a 7-year period independent of potential confounding factors, including long-term diabetes complications (ie, cognitive impairment, depressive symptoms, and cardiovascular diseases). Multiple biological and physiopathological mechanisms support a causal relationship between diabetes and the decline in muscle function, including reduced physical activity, obesity, and traditional microvascular and macrovascular diabetic complications. For example, peripheral neuropathy and peripheral arterial diseases have been suggested to be important determinants of accelerated muscle atrophy and loss of muscle strength in patients with diabetes.^6^ Overweight and obesity are common among older persons with type 2 diabetes, and elevated BMI has been related to increased fat infiltration into the skeletal muscle. Aging, obesity, and increased muscle fat infiltration have been associated with low-grade systemic inflammation, increased oxidative stress, impaired mitochondrial function, and reduced maximal aerobic capacity, linked to age-related decline in muscle mass and strength.^31^ In addition, insulin resistance, the metabolic hallmark of type 2 diabetes and obesity, is associated with muscle wasting, partly due to increased protein degradation and inhibition of protein synthesis.^13^

Our analysis demonstrated that patients with diabetes treated with insulin had a grip strength trajectory similar to those without diabetes, whereas patients with diabetes using oral antidiabetic agents (eg, metformin, sulfonylureas) experienced a significantly steeper decline in muscle strength. In preclinical studies, metformin has been shown to improve skeletal muscle repair by adenosine monophosphate‐activated protein kinase activation. Moreover, this drug might enhance mitochondrial function in skeletal muscle and induce irisin release, an exercise‐induced myokine. Nevertheless, clinical studies investigating the effect of metformin on muscle function are scant and yielded conflicting results. Furthermore, animal studies have suggested that sulfonylureas and glinides might induce muscle atrophy through reduced muscle protein content, muscle weight, and muscle fiber diameter.^32^

On the other hand, several studies have shown that insulin promotes protein synthesis, attenuates protein breakdown, and improves mitochondrial capacity in skeletal muscle.^33^ A recent observational clinical study reported an increased psoas muscle index assessed by computed tomography in middle-aged patients on insulin therapy compared to those on oral antidiabetic drugs and controls.^34^ From this point of view, our study, conducted in a large sample of older people, suggested a potential role of insulin therapy in preserving muscle mass and, more importantly, muscle function.

In agreement with other epidemiological studies, our findings support an inverse and independent relationship between HG strength and incident diabetes over time. Of note, previous studies on this topic were mainly conducted in middle-aged participants.^35^^,^^36^ The only work involving older persons reported a significant association between muscle area and incident diabetes in men but did not observe any association with grip strength.^37^ Our study demonstrated a gradually increased risk of diabetes with lower strength independent of body weight, physical activity level, and baseline glucose level, suggesting reduced muscle strength as a potential risk factor for diabetes and a biomarker for glucose dysregulation in older persons. The association between muscle strength and incident diabetes is not fully understood. Nevertheless, various epidemiological studies demonstrate an independent association between insulin resistance and HG strength in men^38^ and women,^39^ and with gait speed^40^ in men but not in women. Skeletal muscle is the largest insulin-sensitive tissue, accounting for about 80% of glucose uptake under euglycemic hyperinsulinemic conditions. Since skeletal muscle insulin resistance, a fundamental mechanism in the onset of type 2 diabetes, may develop several years before hyperglycemia, it is likely that significantly lower skeletal muscle mass might be the cause of reduced capacity for glucose disposal in older adults with reduced grip strength. Furthermore, age-related changes in skeletal muscle composition and quality, explaining the faster decline in muscle strength compared to muscle mass, are also involved in the pathogenesis of insulin resistance.

In interpreting these findings, some limitations should be considered. First, our study lacks a direct marker of glycemic control. The assessment of glycated hemoglobin would have allowed for a better investigation of the potential role of glycemic control in the pathogenesis of muscle strength decline, but that data were not available for all the study participants. Second, a direct assessment of muscle mass was not performed with the same method in both cohorts. Therefore, we could not appropriately harmonize that parameter and evaluate changes in muscle mass over time, nor measures of strength normalized to muscle mass. Nevertheless, muscle strength is considered a more specific and reliable indicator of muscle health than muscle mass, and normalization for body weight has been previously used in the literature.^14^ Further studies examining the relationship between diabetes and muscle quality (ie, the ratio of muscle strength to muscle mass) are needed in order to confirm our findings and expand the knowledge on this topic. Fourth, muscle strength was assessed only as HG strength. It has been suggested that older patients with diabetes are more likely to experience strength decline in the lower extremities.^41^ However, grip strength is an appropriate measure to identify overall muscle weakness in older adults.^42^ Finally, we did not record the type of diabetes mellitus of the study participants. Although epidemiological data suggest that around 90-95% of diabetes cases in the population are type 2 diabetes (as we can argue for the majority of the study sample), the prevalence of type 1 diabetes is increasing due to the aging of the population and the care improvements.^43^ Therefore, as suggested by some publications, the study of muscle dysfunctions related to type 1 diabetes and possibly modulated by antidiabetic drugs is still worth investigating.^44^

Our study also has notable strengths. Most importantly, the study sample, which included two different population–based cohorts, enhanced the statistical power of the analysis and the external validity of our findings. Second, the prospective design and the large sample size allowed us to make inferences regarding the temporal relationship between time-varying diabetes and muscle strength decline and, simultaneously, between grip strength and incident diabetes. Finally, the inclusion of older people, with a substantial proportion of octogenarians, made it possible to investigate the subgroup of patients with diabetes having the highest risk for muscle impairment and physical disability.

## Conclusions

Handgrip strength assessment is simple, quick, reproducible, low-cost, and, therefore, easy to implement in routine clinical practice and diabetes standard care. A recent randomized clinical study demonstrated that a multicomponent intervention targeting physical frailty and sarcopenia might postpone and reduce the risk of mobility disability in older patients with diabetes.^45^ Screening and periodic assessment of muscle strength may be recommended in people with diabetes, as well as promoting an active lifestyle and a healthy diet. In turn, grip strength evaluation might help identify people at high risk of type 2 diabetes in the general older population, although whether increasing muscle mass and strength would reduce the onset of diabetes has still to be demonstrated.

## Supplementary Material

glag004_Supplementary_Data

## Data Availability

The datasets analyzed in the current study are available from the corresponding author F.R. upon reasonable request.
