# Supplementary material for: The bidirectional relationship between diabetes and poor muscle function in older adults: data from two population-based studies
Source: J Gerontol A Biol Sci Med Sci. 2026 Jan 14;81(4):glag004. doi: 10.1093/gerona/glag004 (PMC13017108; doi:10.1093/gerona/glag004)
Supplement: glag004_Supplementary_Data [file glag004_supplementary_data.zip › SUPPLEMENTARY MATERIAL rev1 clean.docx]

**SUPPLEMENTARY MATERIAL**

**Supplementary Table 1. Comparison of the characteristics of participants included and excluded from the analytical sample**

|  | **Included** | **Excluded** | **P-value** |
| --- | --- | --- | --- |
| n | 3927 | 327 |  |
| Age (years) | 75.46 (7.45) | 83.80 (7.63) | <0.001 |
| Female sex | 2302 (58.6) | 208 (63.6) | 0.088 |
| Educational level |  |  | 0.247 |
| None or elementary | 3317 (84.5) | 281 (87.3) |  |
| Middle or High school | 528 (13.4) | 33 (10.2) |  |
| University or above | 82 (2.1) | 8 (2.5) |  |
| Civil status |  |  | <0.001 |
| Never married, single | 299 (7.6) | 37 (11.4) |  |
| Partnered | 2126 (54.2) | 98 (30.2) |  |
| Separated/divorced | 24 (0.6) | 2 (0.6) |  |
| Widowed | 1475 (37.6) | 176 (54.2) |  |
| Smoking habits |  |  | <0.001 |
| Never smokers | 2367 (60.3) | 228 (72.4) |  |
| Former smokers | 1177 (30.0) | 63 (20.0) |  |
| Current smokers | 381 (9.7) | 24 (7.6) |  |
| Alcohol consumption |  |  | <0.001 |
| No | 2298 (58.7) | 230 (72.8) |  |
| Mild to moderate | 903 (23.0) | 64 (20.3) |  |
| Heavy | 717 (18.3) | 22 (7.0) |  |
| Low physical activity | 1194 (30.7) | 238 (75.6) | <0.001 |
| Body Mass Index (kg/m2) | 27.57 (4.46) | 25.43 (4.34) | <0.001 |
| ADL score | 5.29 (1.20) | 3.19 (2.50) | <0.001 |
| IADL score | 6.34 (2.19) | 3.06 (3.12) | <0.001 |
| MMSE | 23.85 (5.32) | 13.76 (10.61) | <0.001 |
| Depressive symptoms | 1217 (33.0) | 66 (43.7) | 0.008 |
| Arterial hypertension | 2790 (71.1) | 177 (54.6) | <0.001 |
| Cardiovascular diseases | 1066 (27.1) | 161 (49.2) | <0.001 |
| Previous hip fracture | 134 (3.4) | 31 (9.5) | <0.001 |
| Osteoarthritis | 1251 (31.9) | 119 (36.4) | 0.104 |
| COPD | 421 (10.7) | 45 (13.8) | 0.110 |
| Cancer | 298 (7.6) | 22 (6.7) | 0.645 |
| Diabetes mellitus | 607 (15.5) | 54 (16.5) | 0.669 |

*Notes*. Missing values in educational status (n=5), civil status (n=3), smoking habits (n=14), alcohol consumption (n=20), depressive symptoms (n=419), ADL (n=16), IADL (n=16), BMI (n=365), MMSE (n=71), arterial hypertension (n=5), hip fracture history (n=6), cancer (n=2), COPD (n=2). *Abbreviations*: ADL, activities of daily living; IADL, instrumental activities of daily living; MMSE, mini-mental state examination; COPD, chronic obstructive pulmonary disease.

**Supplementary Table 2. Comparison of the characteristics of participants stratified by original cohort**

|  | **InCHIANTI** | **Pro.V.A.** | **P-value** |
| --- | --- | --- | --- |
| n | 1018 | 2909 |  |
| Age (years) | 74.54 (7.06) | 75.78 (7.55) | <0.001 |
| Female sex | 577 (56.7) | 1725 (59.3) | 0.155 |
| Educational level |  |  | <0.001 |
| None or elementary | 754 (74.1) | 2563 (88.1) |  |
| Middle or High school | 228 (22.4) | 300 (10.3) |  |
| University or above | 36 (3.5) | 46 (1.6) |  |
| Civil status |  |  | <0.001 |
| Never married, single | 72 (7.1) | 227 (7.8) |  |
| Partnered | 625 (61.4) | 1501 (51.6) |  |
| Separated/divorced | 9 (0.9) | 15 (0.5) |  |
| Widowed | 312 (30.6) | 1163 (40.0) |  |
| Missing | 0 (0.0) | 2 (0.1) |  |
| Smoking habits |  |  | 0.072 |
| Never smokers | 596 (58.5) | 1771 (60.9) |  |
| Former smokers | 303 (29.8) | 874 (30.0) |  |
| Current smokers | 119 (11.7) | 262 (9.0) |  |
| Missing | 0 (0.0) | 2 (0.1) |  |
| Alcohol consumption |  |  | <0.001 |
| No | 275 (27.0) | 2023 (69.5) |  |
| Mild to moderate | 379 (37.2) | 524 (18.0) |  |
| Heavy | 357 (35.1) | 360 (12.4) |  |
| Missing | 7 (0.7) | 2 (0.1) |  |
| Low physical activity | 202 (19.8) | 992 (34.6) | <0.001 |
| Body Mass Index (kg/m^2^) | 27.46 (4.08) | 27.61 (4.58) | 0.373 |
| ADL score | 5.87 (0.61) | 5.09 (1.29) | <0.001 |
| IADL score | 7.27 (1.74) | 6.01 (2.23) | <0.001 |
| MMSE | 24.80 (4.11) | 23.51 (5.64) | <0.001 |
| Depressive symptoms |  |  | <0.001 |
| No | 668 (65.6) | 1799 (61.8) |  |
| Yes | 322 (31.6) | 895 (30.8) |  |
| Missing | 28 (2.8) | 215 (7.4) |  |
| Arterial hypertension | 628 (61.7) | 2162 (74.4) | <0.001 |
| Cardiovascular diseases | 230 (22.6) | 836 (28.7) | <0.001 |
| Previous hip fracture | 32 (3.1) | 102 (3.5) | 0.646 |
| Osteoarthritis | 314 (30.8) | 937 (32.2) | 0.444 |
| COPD | 77 (7.6) | 344 (11.8) | <0.001 |
| Cancer | 64 (6.3) | 234 (8.0) | 0.079 |
| Diabetes mellitus | 128 (12.6) | 479 (16.5) | 0.004 |
| Handgrip (kg) | 28.57 (11.95) | 25.98 (9.51) | <0.001 |

*Abbreviations*: ADL, activities of daily living; IADL, instrumental activities of daily living; MMSE, mini-mental state examination; COPD, chronic obstructive pulmonary disease.

**Supplementary Table 3. Linear mixed model for the association between diabetes and changes in handgrip over the follow-up by sex**

|  | **β coefficient (95%Confidence interval), *p-value*** | | | | | |
| --- | --- | --- | --- | --- | --- | --- |
|  | **Model 1** | **P** | **Model 2** | **P** | **Model 3** | **P** |
| **Men (n=1625)** |  |  |  |  |  |  |
| Diabetes (intercept, T0) | -0.13  (-1.15, 0.90) | 0.810 | -0.51  (-1.52 – 0.49) | 0.318 | -0.37  (-1.37 – 0.63) | 0.469 |
| Diabetes*T1 | -0.95  (-2.02, 0.13) | 0.084 | -0.81  (-1.88 – 0.26) | 0.137 | -0.78  (-1.85 – 0.28) | 0.150 |
| Diabetes*T2 | -0.77  (-2.18, 0.64) | 0.285 | -0.59  (-1.99 – 0.81) | 0.413 | -0.49  (-1.89 – 0.91) | 0.494 |
| **Women (n=2302)** |  |  |  |  |  |  |
| Diabetes (intercept, T0) | -0.21  (-0.82, 0.41) | 0.511 | 0.23  (-0.38 – 0.84) | 0.465 | 0.31  (-0.30 – 0.92) | 0.316 |
| Diabetes*T1 | -0.37  (-1.06, 0.31) | 0.283 | -0.54  (-1.22 – 0.15) | 0.124 | -0.49  (-1.17 – 0.19) | 0.161 |
| Diabetes*T2 | -0.70  (-1.57, 0.18) | 0.121 | -0.92  (-1.80 – -0.04) | **0.042** | -0.93  (-1.80 – -0.05) | **0.039** |

Model 1 is adjusted for study cohort and age. Model 2 is also adjusted for educational level, smoking habits, alcohol consumption, low physical activity, Mini-Mental State Examination, osteoarthritis, cancer, chronic obstructive pulmonary disease, and depressive symptoms. Model 3 is also adjusted for cardiovascular diseases (time-varying variable). *Notes*: Diabetes status is included in the model as a time-varying variable. Bold font indicates significant P-values (<0.05).

**Supplementary Table 4. Linear mixed model for the association between diabetes and changes in handgrip over the follow-up by study cohort**

|  | **β coefficient (95%Confidence interval), *P-value*** | | |
| --- | --- | --- | --- |
|  | **Pro.V.A. study** | **InCHIANTI study** |  |
| Diabetes (intercept, T0) | 0.44 (-0.12 – 1.01), P=0.126 | -0.72 (-2.09 – 0.65), P=0.306 |  |
| Diabetes*T1 | -0.51 (-1.09 – 0.06), P=0.081 | -0.63 (-2.13 – 0.87), P=0.412 |  |
| Diabetes*T2 | -0.45 (-1.31 – 0.41), P=0.302 | -0.40 (-1.95 – 1.15), P=0.614 |  |

Model adjusted for age, sex, educational level, smoking habits, alcohol consumption, low physical activity, Mini-Mental State Examination, osteoarthritis, cancer, chronic obstructive pulmonary disease, depressive symptoms, and cardiovascular diseases (time-varying variable). *Notes*: Diabetes status is included in the model as a time-varying variable. The analysis involved 2836 Pro.V.A. participants and 1011 InCHIANTI participants with complete data on the variables included in the model.

**Supplementary Table 5. Cox regression for the association between the baseline handgrip/body weight ratio and incident diabetes over the follow-up by study cohort**

|  | **Hazard Ratio of diabetes (95% Confidence Interval),**  ***P-value*** | |
| --- | --- | --- |
|  | **Pro.V.A. study** | **InCHIANTI study** |
| *Per each 1-SD HG/weight increase* | 0.77 (0.63 – 0.94), P=0.009 | 0.84 (0.62 – 1.15), P= 0.28 |

Model adjusted for study cohort, age, sex, educational level, smoking habits, alcohol consumption, low physical activity, depressive symptoms, and cardiovascular diseases at baseline. *Notes*: A 1-SD difference in the HG/ body weight ratio corresponded to 0.13 in males, and 0.11 in females. *Abbreviations*: HG, handgrip; SD, standard deviation. The analysis involved 2313 Pro.V.A. participants (n=133 incident cases of diabetes) and 755 InCHIANTI participants (n=38 incident cases of diabetes) free from diabetes at baseline and with complete data on the variables included in the model.
